# An Anomalous Disease of the Tongue and Feet

**Published:** 1846-01

**Authors:** G. N. Fitch

**Affiliations:** Logansport, Indiana


ILLINOIS

MEDICAL & SURGICAL JOURNAL.

VOL. II. JANUARY, 1846. NO. 10.

PROF. BLANEY: After the lapse of a year you have my promised continuation of the article on "an Anomalous disease of the Tongue and Feet," published in the February No. of the Journal, 1845. Constant professional engagement is my only excuse for this long delay. G. N. FITCH.

In treating it while confined to the tongue and mouth, the ordinary collutories for other forms of glossitis, were at first prescribed, as borate of soda, alum, sulph. zinc, nitrate arg., &c., in solution, together with diluted muriatic, and sulphuric acid, and all with about equal success. A few milder cases appeared to be benefited by these remedies, though subsequent experience convinced me that the recovery was, except in very few instances, in no wise attributable to the remedies, but rather in spite of them. If any one of the above mentioned was decidedly beneficial, it was the dilute muriatic acid. Laxative doses of the sulph. magnesia were ordered with all these local means, and their continued use appeared productive of relief in all cases, to some extent. No mucilaginous wash procured a moment's cessation of the intense burning pain, though the common slippery elm, (*ulmus flava*) when chewed was very grateful; never productive of pain, and in many instances, entirely allaying it for quite an interval. Emetics were prescribed, as I first flattered myself with some success; but cures with them were soon found to be like those with the enumerated local remedies,—spontaneous. Mercurials were used, and pushed in some cases to ptyalism, with no other result than an aggravation of pain and other symptoms. The local means upon which I was compelled to rely, and from which the greatest benefit was derived, in the greatest number of cases, were, equal parts, by weight, of borate of soda and ext. catechu finely pulv. and intimately mixed, thrown into the mouth in small quantities, or mixed with syrup or honey, in the proportion of ℥ss of the compound to ℥ij of the syrup, and a tea spoonful taken into the mouth, and retained ten or fifteen minutes. This remedy failing, I used, with decided benefit, collutories, composed of alcoholic solution of creasote. By the persevering use of one or both of these means, conjoined with saline laxatives, (sulph. magnesia) the symptoms were much ameliorated, and many unequivocal cures obtained. In some cases they failed, as did every other means to which resort was had, and the disease either attacked the feet, or exhausted itself upon the tongue and mouth, after producing considerable emaciation and debility.

After the feet were attacked, the "burning" induced some to bathe them in cold water. If any relief was obtained by this process, it was but temporary, and at the expense, usually, of a subsequent increase of suffering. In some instances violent cramp of the lower extremities; in others, severe gastralgia, or nausea and vomiting, or headache immediately followed the application of cold. Relief in many cases was derived from cupping. Blisters were applied to the ancles and superior parts of the feet, but with doubtful efficacy. Next to cups, and indeed preferable to them in milder cases, was a tepid nitro-muriatic acid foot bath, sufficiently active to soon corrugate the skin, and produce a decided "tingling" sensation. A wash of the alcoholic solution of creasote was found highly useful here likewise. Opiates were administered in this, as likewise in the lingual form of the disease, and procured as usual temporary exemption from intense suffering, but exerted no permanent beneficial influence. Many who suffered under this form, (in the feet) during the spring months derived marked relief from placing their feet for a length of time (one to three hours) in soft, newly ploughed land. Laborers, who were unable to place their feet upon the floor or unbroken surface of the ground, without an aggravation of pain, have, after short persistance in their efforts, followed the plough, walking in the new furrow, all day, and in a few days, been by these means alone measurably cured.

It will be perceived that the treatment, so far, has been altogether experimental and empirical. Experience has justified a subsequent resort to but few of the many remedies tried. Other physicians on the upper Wabash have seen the disease.

Perhaps their experience may have suggested some more rational and successful method of treatment than the one I have adopted; if so a publication of their views and treatment will entitle them to the gratitude, not only of the profession, but of the many yet suffering from the disease.

Logansport, Indiana, January, 1846.

